# Four new species of *Lesticus* (Carabidae, Pterostichinae) from China and supplementary comments on the genus

**DOI:** 10.3897/zookeys.782.27187

**Published:** 2018-08-27

**Authors:** Pingzhou Zhu, Hongliang Shi, Hongbin Liang

**Affiliations:** 1 College of Biological Sciences and Technology, Beijing Forestry University, Beijing 100083, China; 2 College of Forestry, Beijing Forestry University, Beijing 100083, China; 3 Key Laboratory of Zoological Systematics and Evolution, Institute of Zoology, Chinese Academy of Sciences, Beijing 100101, China

**Keywords:** Trigonostomina, beetle, key, new species, endophallus, character evolution

## Abstract

The genus *Lesticus* in China was studied, with descriptions of four new species: *L.auripennis***sp. n.** (Guangdong: Nanling, 24.93°N, 112.09°E); *L.bii***sp. n.** (Xizang: Mêdog, 29.32°N, 95.34°E); *L.xiaodongi***sp. n.** (Yunnan: Yingjiang, 24.71°N, 97.58°E); and *L.violaceous***sp. n.** (Yunnan: Yingjiang, 24.71°N, 97.58°E). One new synonym is proposed: *L.dubius* Dubault, Lassalle & Roux, is a junior synonym of *L.solidus* Roux & Shi. An improved key and a catalogue accommodating all known Chinese *Lesticus* species are provided. Species relationships and the evolution of endophallic and other characters are preliminarily discussed.

## Introduction

*Lesticus* Dejean belongs to the subtribe Trigonostomina (Carabidae: Pterostichinae) which includes six genera having an Oriental-Australasian distribution. This subtribe can be easily recognized by the very short and wide mentum tooth. *Lesticus* can be distinguished from other genera in the subtribe by the following character combination: first antennomere shorter than the sum of the following three segments; long antennae reaching the elytral base; labrum apex straight with six equally placed setae; terminal labial palpomere truncated at the apex; mentum not notably shortened; scutellar striae complete.

In China, species diversity of *Lesticus* is highest in tropical regions, especially Yunnan Province, but one species (*L.magnus*) is primarily distributed in northern China. Some species are widely distributed (e.g. *L.auricollis*) or locally abundant (e.g. *L.rotundatus*), while others are rare within a very narrow distribution (e.g. *L.ater*). *Lesticus* beetles tend to prefer mountain tropical or subtropical primary forests in China, having mainly nocturnal and ground-dwelling habits. Most specimens were collected under shelters, by pitfall trap, or on dirt paths during the night.

Before the present investigation, a total of 15 *Lesticus* species had been recorded in China (Roux et al. 2016), with most of them described in the past 10 years. But species determination is still difficult because some species are superficially similar to others, both from external and male genital (except endophallic) characters, and others are very rare in collections. Moreover, in the published keys (Roux and Shi 2011, Roux et al. 2016), some distinguishing characters were infraspecifically variable (e.g. pronotal basal punctations, body size) or ambiguously defined (e.g. pronotal shape, dorsal color).

In recent expeditions to southern China (W. Yunnan, S. Xizang, Guangdong), four very rare and narrowly distributed new species of *Lesticus* were collected. Aside from these, more new species are expected to be discovered in southern China, because some undetermined female specimens were present as well. When studying the new species, we found that the male endophallic characters have very important taxonomical value, both for systematics and species identification. Thus, we studied the male endophallus for all available Chinese species and a few from other countries. As a result, part of the endophallic character evolution in *Lesticus* was revealed, as well as some of the species relationships. Moreover, we found that the male holotype of *L.dubius* is very similar to, and sympatric with, *L.solidus* (only females known), and their main differences (elytra more expanded in *L.solidus*) are in fact sexual differences.

The primary purpose of the present paper is to provide an improved key and checklist with geographical distributions for all known Chinese species, to describe four new species, and to propose one new synonym. Thus, the known Chinese *Lesticus* fauna now totals 18 species with 14 of them endemic to China. For all the new species, complete descriptions, illustrations and distribution maps are provided, particularly with the addition of endophallic characters. Additionally, the evolution of endophallic characters and species relationships of *Lesticus* are briefly discussed, with four main endophallic types defined, and the present three subgenera of *Lesticus* rejected. An improved catalogue of *Lesticus* from China with new province records is presented as well.

## Materials and methods

This work was based primarily on examination of specimens from China. The majority of specimens examined, including all types of new species, are deposited in the collection of the Institute of Zoology, Chinese Academy of Science, Beijing, China (**IZAS**). Collections cited in the present paper are indicated by the following abbreviations:

**CCCC** Collection of Changchin Chen, Tianjin, China

**CDW** Collection of David Wrase, Berlin, Germany

**MNHN** Muséum National d’Histoire Naturelle, Paris, France

**MNHU** Museunm für Naturkunde der Humboldt- Universität zu Berlin, Berlin, Germany

**NHMB** Naturhistorisches Museum, Basel, Switzerland

**NMNS** National Museum of Natural Science, Taiwan, China

**NMPC** Národní Muzeum Přírodovědecké Muzeum, Prague, Czech Republic

**SCAU** South China Agriculture University, Guangzhou, China

**ZMUM** Zoological Museum, Moscow State University, Moscow, Russia

The length of metepisternum was measured along its outer margin; the basal width was measured along its oblique basal margin (Figs 7, 18). When describing the endophallus, all lobes were named based on their location in the basic type (Type 1) as defined in the Discussion; for the derived types, lobes were named according to homology inferences rather than actual locations. Other terms used and methods of measurement, preparation of figures, dissection and endophallus everting procedures mainly follow our previous work (Shi and Liang 2015).

### Key to Chinese species of *Lesticus*

**Table:** 

1	Metepisternum short and wide, length less than or subequal to its basal width (Fig. 9)	**2**
–	Metepisternum long and narrow, length much greater (more than 1.3 times) than its basal width (Fig. 23)	**7**
2	Odd intervals raised, distinctly wider than even ones; S.E. Xizang	***L.bii* sp. n.**
–	Odd intervals normal, same level and width as even ones	**3**
3	Propleuron, metepisternum and pronotal basal fovea completely glabrous; lateral margins straight before posterior angles; apex of median lobe strongly truncated; S. Yunnan	***L.ater* Roux & Shi**
–	Metepisternum with at least a few coarse punctations; propleuron punctated or glabrous; pronotal basal rugose or punctated, although sometimes very finely; lateral margins slightly sinuate before posterior angles; apex of median lobe rounded or less truncated	**4**
4	Pronotal basal fovea very shallow, with very fine wrinkles and punctures; median lobe of aedeagus more or less expanded ventrally; other provinces	**5**
–	Pronotal basal fovea moderately deep, distinctly punctated; median lobe of aedeagus straight ventrally; Fujian	**6**
5	Dorsal side almost uniformly black, elytra black or with very weak purple metallic lustre; median lobe of aedeagus strongly expanded ventrally; Yunnan, Guangxi	***L.perniger* Roux & Shi**
–	Dorsal side distinctly bicolored: head and pronotum black, elytra with strong metallic lustre, purple or green; median lobe of aedeagus slightly expanded ventrally; Guangdong	***L.auripennis* sp. n.**
6	Pronotal basal fovea with coarser punctures; aedeagus apex more rounded; northern Fujian	***L.fukiensis* Jedlička**
–	Pronotal basal fovea with finer punctures; aedeagus apex slightly truncated; southern Fujian	***L.wrasei* Dubault et al.**
7	Pronotum somewhat metallic, green, cupreous or purple	**8**
–	Pronotum completely black, without metallic lustre	**15**
8	Propleuron and metepisternum glabrous or with sparse and fine punctures	**9**
–	Propleuron and metepisternum coarsely and densely punctated	**10**
9	Pronotum with strong metallic lustre; basal fovea nearly glabrous, with very fine punctures or wrinkles at most; smaller species, 21–23 mm	***L.chalcothorax* (Chaudoir)**
–	Pronotum with very faint metallic lustre; basal fovea coarsely punctated; larger species, 24–29 mm	***L.praestans* (Chaudoir)**
10	Basal fovea densely punctated, punctures also present on middle region of pronotum base; median lobe of aedeagus markedly expanded ventrally	***L.rotundatus* Roux & Shi**
–	Pronotum basal fovea glabrous or punctated, without puncture on middle region of pronotum base; median lobe of aedeagus nearly straight ventrally	**11**
11	Pronotum lateral margins somewhat sinuate before posterior angles; posterior angles forming an obtuse angle; Yunnan	**12**
–	Pronotum lateral margins completely straight before posterior angles; posterior angles obtusely rounded; other provinces	**13**
12	Pronotum lateral margins strongly sinuate before posterior angles; posterior angles pointed a little outwards; pronotum metallic green, elytra blue	***L.xiaodongi* sp. n.**
–	Pronotum lateral margins slightly sinuate before posterior angles; posterior angles not pointed; pronotum metallic bluish violet, elytra violet	***L.violaceous* sp. n.**
13	Pronotal basal fovea distinctly punctated; pronotum green; Guangxi	***L.deuvei* Dubault & Roux**
–	Pronotal basal fovea nearly glabrous, with very fine punctures or wrinkles at most; pronotum cupreous-green; other provinces	**14**
14	Elytral striae punctate relatively coarser; median lobe of aedeagus indistinctly constricted at basal third, apical orifice larger; China Mainland	***L.auricollis* Tschitschérine**
–	Elytral striae punctated relatively finer; median lobe of aedeagus markedly constricted at basal third, apical orifice smaller; Taiwan	***L.taiwanicus* Roux & Shi**
15	Pronotal basal fovea glabrous, with very fine wrinkles at most; propleuron anterior half with sparse punctures	**16**
–	Pronotal basal fovea with dense punctures and wrinkles; propleuron anterior half with dense punctures	**17**
16	Elytra completely black, not metallic; pronotal lateral margins slightly sinuate before posterior angles; posterior angles pointed a little outwards; smaller species, 25–27 mm; Yunnan	***L.tristis* Roux & Shi**
–	Elytra with very faint purple metallic lustre; pronotal lateral margins straight before posterior angles; posterior angles completely rounded; larger species, 28–32 mm; Guangxi	***L.solidus* Roux & Shi**
17	Pronotal basal fovea deeper, with coarse punctures; lateral margin less sinuate before posterior angles; smaller species, 20–27 mm; China Mainland, Korea, Japan	***L.magnus* (Motschulsky)**
–	Pronotal basal fovea shallower, with fine punctures; lateral margin more sinuate before posterior angles; larger species, 27–30 mm; Taiwan	***L.sauteri* Kuntzan**

#### Lesticus auripennis sp. n.

Taxon classificationAnimaliaColeopteraCarabidae

http://zoobank.org/1C717FB3-A47E-4CB3-A620-D0DD18202CE4

Figs 1–4

##### Type locality.

China, Guangdong: Nanling (24.93°N, 112.99°E), altitude 1587 m.

##### Type material.

**Holotype**: male (IZAS), body length = 20.4 mm, board mounted, genitalia preserved in 100% ethanol in microvial pinned under specimen, “China, Guangdong / Ruyuan, Nanling / pitfall trap / 24.932039N, 112.996099E”; “1587m, 2017.VI.4-7 / Liu Y. Z. & Yu S. P. lgt., / Insititude of Zoology., CAS.”; “HOLOTYPE♂ / *Lesticusauripennis* sp. n. / des. ZHU & SHI, 2018” [red label]. **Paratypes** (two males and six females): one male and one female (IZAS), the same data as holotype but labeled as paratype. One female (IZAS), “China, Guangdong / Ruyuan, Nanling / pitfall trap / 24.93077N, 112.994692E”; “1708m, 2017.VI.4-7 / Liu Y. Z. & Yu S. P. lgt., / Insititude of Zoology., CAS.”; “PARATYPE♂ / *Lesticusauripennis* sp. n. / des. ZHU & SHI 2018” [red label]. One male and four females (SCAU), “Guangdong, Nanling 2008 / 24.9284N, 113.0163E / 1035m, pitfall trap, Gao Lei / South China Agriculture University”; “PARATYPE♂ / *Lesticusauripennis* sp. n. / des. ZHU & SHI 2018” [red label].

**Figures 1–4.**
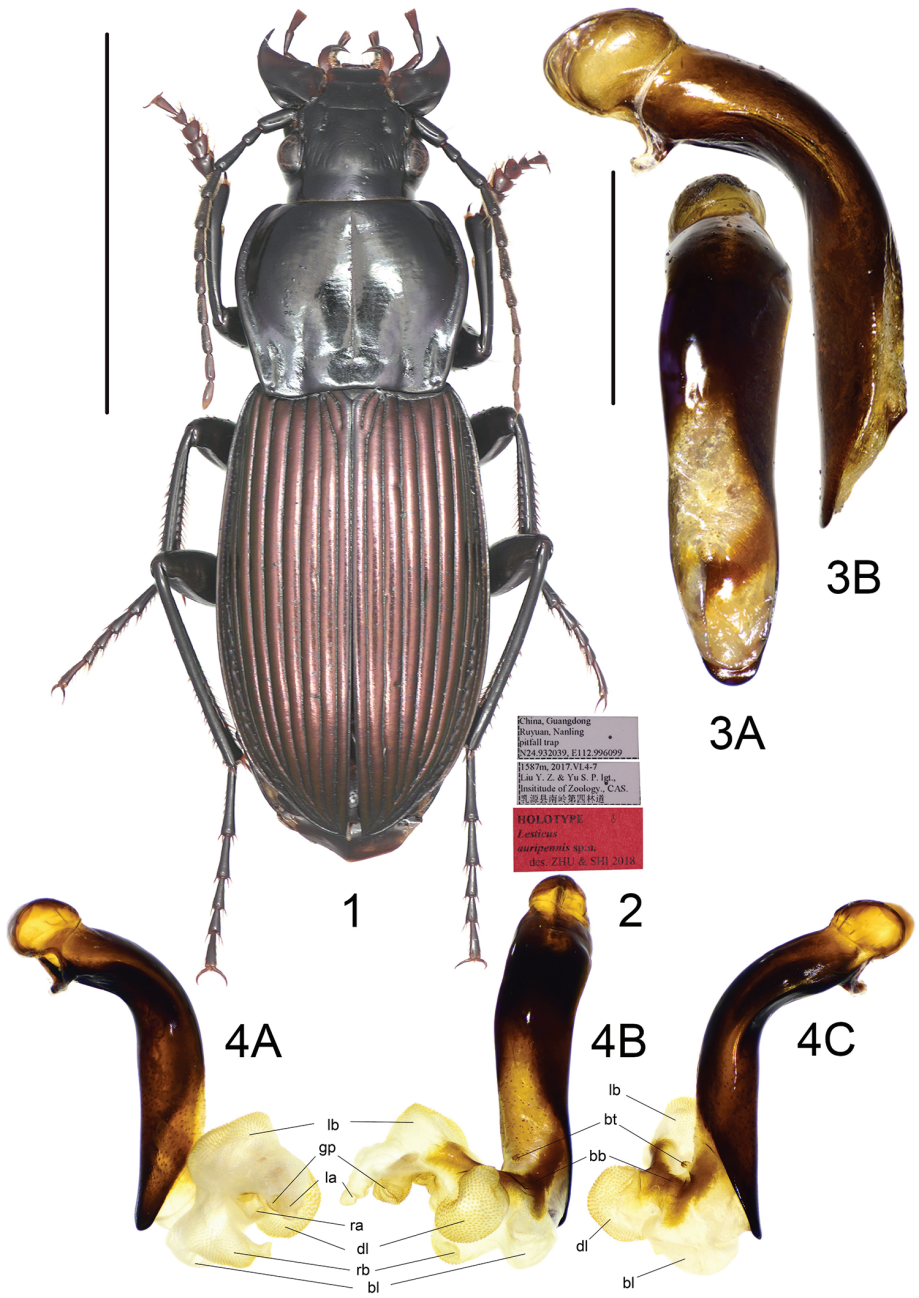
*Lesticusauripennis* sp. n. **1** habitus of holotype (male, Guangdong, IZAS), scale bar: 10.0 mm **2** labels of holotype **3** median lobe of aedeagus (holotype) **A** dorsal view **B** left-lateral view, scale bar: 2.0 mm **4** endophallus (holotype) **A** left lateral view **B** dorsal view **C** right lateral view.

##### Diagnosis.

Pronotum completely black; elytra metallic green or purple; pronotal lateral margins slightly sinuate before posterior angles; pronotal basal fovea almost glabrous, with restricted and very fine punctures only; metepisternum short and wide, length subequal to its basal width, with coarse punctures.

##### Comparisons.

The new species is the only one among all Chinese *Lesticus* species with black pronotum and distinctly metallic elytra. *Lesticusauripennis* sp. n. is most similar to *L.perniger* and *L.wrasei* sharing the short, wide metepisternum with coarse punctures; pronotal lateral margins slightly sinuate before posterior angles; pronotal basal fovea shallowly incised; elytral intervals normal. The new species is distinguishable from the latter two species by elytra metallic color, restricted and very fine pronotal basal foveal punctures and a different shape of male genitalia. Median lobe of aedeagus of the new species is very similar to that of *L.wrasei*, but different in: (1) ventral margin slightly expanded near middle (versus completely straight); (2) apex a little less truncated; (3) in dorsal view, left margin slightly sinuate near middle (versus evenly curved).

##### Description.

Body length 19.5–23.8 mm, elytra’s greatest width 7.2–8.7 mm, both sexes with similar body forms. Head, pronotum, and appendage black and shining; maxillae, labial and maxillary palpomeres, lateral sides of labrum and terminal tarsomeres reddish brown; apical half of terminal palpomere yellow; elytra with strong metallic lustre, usually purple, in some individuals green; ventral side black. Head and pronotum with isodiametric microsculpture and minute punctures; elytra with isodiametric microsculpture.

Head glabrous, without coarse punctures; short and deep frontal depressions extended posterad to middle of eyes, with five to seven shallow longitudinal wrinkles behind frontal depression; anterior margin of labrum slightly emarginate; temporae slightly tumid behind eyes; antennal apex reaching elytra basal tenth.

Pronotum wider than head, PW/HW = 1.49–1.54, a little transverse, PW/PL = 1.24–1.38; pronotum widest near middle. Lateral margins not crenulate, curved in middle, slightly sinuate before posterior angles; posterior angles obtuse, apex rounded, not pointed outwards; posterior margin a little greater than anterior margin, extended slightly backward on each side. Median line shallow but distinct, not reaching posterior margin; disc almost glabrous, with a few shallow transverse wrinkles along median line at most. Basal fovea shallow but well defined; inner groove longer, approximately one-third length of pronotum; outer groove shorter, approximately one-fifth length of pronotum; basal foveal area with very fine punctures and wrinkles along inner and outer grooves, glabrous between inner and outer grooves.

Elytra oviform, EL/EW = 1.39–1.69, gradually widened to apex, widest at posterior third approximately; basal ridge complete, gradually curved, forming a distinct obtuse angle with elytral lateral margin, humeral teeth not pointed. Intervals barely convex; striae deeply incised, with very fine and sparse punctures alongside; scutellar stria short, apex free; parascutellar pore present; third interval with three setigerous pores: first one adjacent to third stria, the other two generally close to second stria (in a male paratype, third pore close to third stria); umbilicular series on ninth interval composed of 20–22 pores evenly spaced. Hind wings very small.

Ventral side: propleuron glabrous, without puncture or wrinkle; mesopleuron with a few coarse punctures on anterior half; metepisternum short and wide, length subequal to its basal width, with sparse and coarse punctures, usually 10–20, sometimes fewer; abdominal sterna glabrous on median portion, with few coarse punctures on lateral sides of sternum II and sometimes also sternum III, and very fine wrinkles on lateral sides of all sterna.

Legs: basal three metatarsomeres with distinct carina along almost full length of outer surface, fourth metatarsomeres with weaker carina only near base; fifth tarsomeres with 3–4 pairs of spines ventrally.

Male genitalia: median lobe of aedeagus with apical orifice opened dorsally; in lateral view, ventral margin slightly expanded in middle, apical portion straight, turned neither ventrally nor dorsally, basal portion slightly narrowed; in dorsal view, apical lamella very short, length approximately one-fourth basal width, apex rounded, slightly truncated; apical portion straight, oriented to neither left nor right. Endophallus (Fig. 4) short, extending to dorsal-left, major portion of endophallus on left-dorsal side of aedeagus when everted; gonopore (**gp**, gonopore lobe folded in Fig. 4) located at a little before apical lamella, oriented to aedeagal apex. Basal tubercle (**bt**) typical of genus; basal band (**bb**) elongated, extended from apical orifice to base of **lb**. Six distinct lobes recognized: dorsal lobe (**dl**) very large and rounded, on right-dorsal surface; one additional basal lobe (**bl**) present on basal-ventral surface, close to **dl**, large and rounded, placed near apical lamella, decorated with very fine and sparse scales; right basal lobe (**rb**) a little smaller than **dl**, close to **bl**, apex sharply pointed, apex with dense and fine scales; right apical lobe (**ra**) on ventral side of **gp**, small, decorated with fine scales; left basal lobe (**lb**) a little larger than **dl**, on dorsal-apical surface, apex with dense scales; left apical lobe (**la**) small, close to **lb**, apex sharply pointing out, decorated with fine scales.

##### Distribution.

**Map 1.**
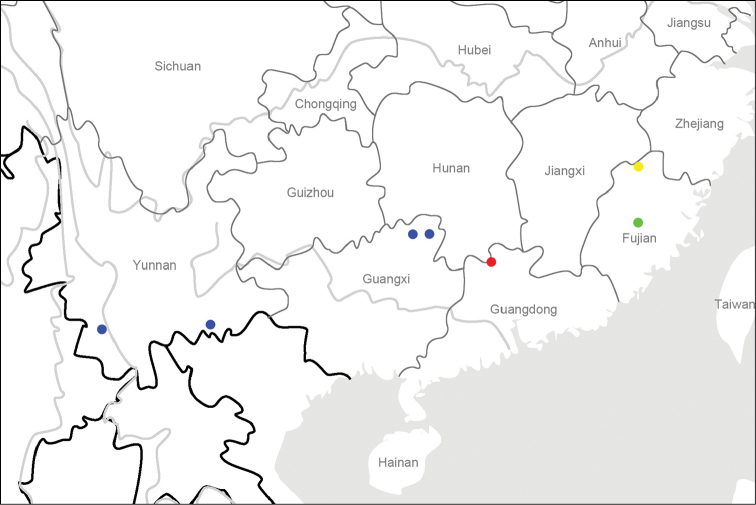
Distribution map for *L.auripennis* sp. n. and allied species: *L.auripennis* sp. n. (red); *L.fukiensis* Jedlicka (yellow); *L.wrasei* Dubault, Lassalle & Roux (green); *L.perniger* Roux & Shi (blue).

This species is known only in Nanling, Guangdong (Map 1). The three localities of the type series are very close together.

##### Etymology.

The name “*auripennis*” is from Latin, “aur-” meaning colored and “pennis” meaning wing, referring to the elytra. This species is named for its distinctly metallic elytra.

##### Affinities.

*Lesticusauripennis* sp. n. is close to *L.perniger*, *L.ater*, *L.wrasei*, and *L.fukiensis* among Chinese *Lesticus* fauna, sharing their short, wide metepisterna and similar shapes of the pronotum. Outside China, there are two other species with these characters: *L.ornatus* Dubault, Lassalle & Roux (Chiang Mai, Thailand) and *L.restrictus* Dubault, Lassalle & Roux (Shan State, Myanmar). These seven species could be recognized as one species group, which can be distinguished from other species of *Lesticus* by: (1) short, wide metepisternum, length less than or subequal to its basal width; (2) elytra third interval with two or three setigerous pores; (3) pronotum completely black, with no trace of metallic; (4) pronotum subquadrate or a little cordifrom; (5) relatively small body size, generally less than 24 mm.

#### Lesticus bii sp. n.

Taxon classificationAnimaliaColeopteraCarabidae

http://zoobank.org/392DF27B-155F-4FA6-B8EE-887727DCB972

Figs 5–9

##### Type locality.

China, Xizang: Mêdog (ca. 29.32°N, 95.34°E), altitude 1500–1900 m.

##### Type material.

**Holotype**: male (IZAS), body length = 17.8 mm, board mounted, genitalia dissected and glued on plastic film pinned under specimen, “Xizang, Mêdog, / 1500–1900m, 2013. / VIII.20, Bi Wenxuan”; “HOLOTYPE ♂ / *Lesticusbii* sp.n. / des. ZHU & SHI 2018” [red label]. **Paratypes** (one male and three females): one male (IZAS), “Xizang, Nyingchi, Mêdog Pari village / 1807m / 2014-VIII-9 / along road during day, Yang Xiaodong Leg. / 14Y0437 CCCC”; “PARATYPE ♂ / *Lesticusbii* sp.n. / des. ZHU & SHI 2018” [red label]. Two females (CCCC), “Xizang, Nyingchi, Mêdog / 1559m / 2016-VIII-5 / along road during night, Yang Xiaodong Leg. / 16Y CCCC”; “PARATYPE ♂ / *Lesticusbii* sp.n. / des. ZHU & SHI 2018” [red label]. One female (IZAS), “China Xizang Mêdog / Phomshen village 1850m / 2016-VII-10 / light trap, Lu Yanquan Leg. / CCCC”; “PARATYPE ♂ / *Lesticusbii* sp.n. / des. ZHU & SHI 2018” [red label].

**Figures 5–9.**
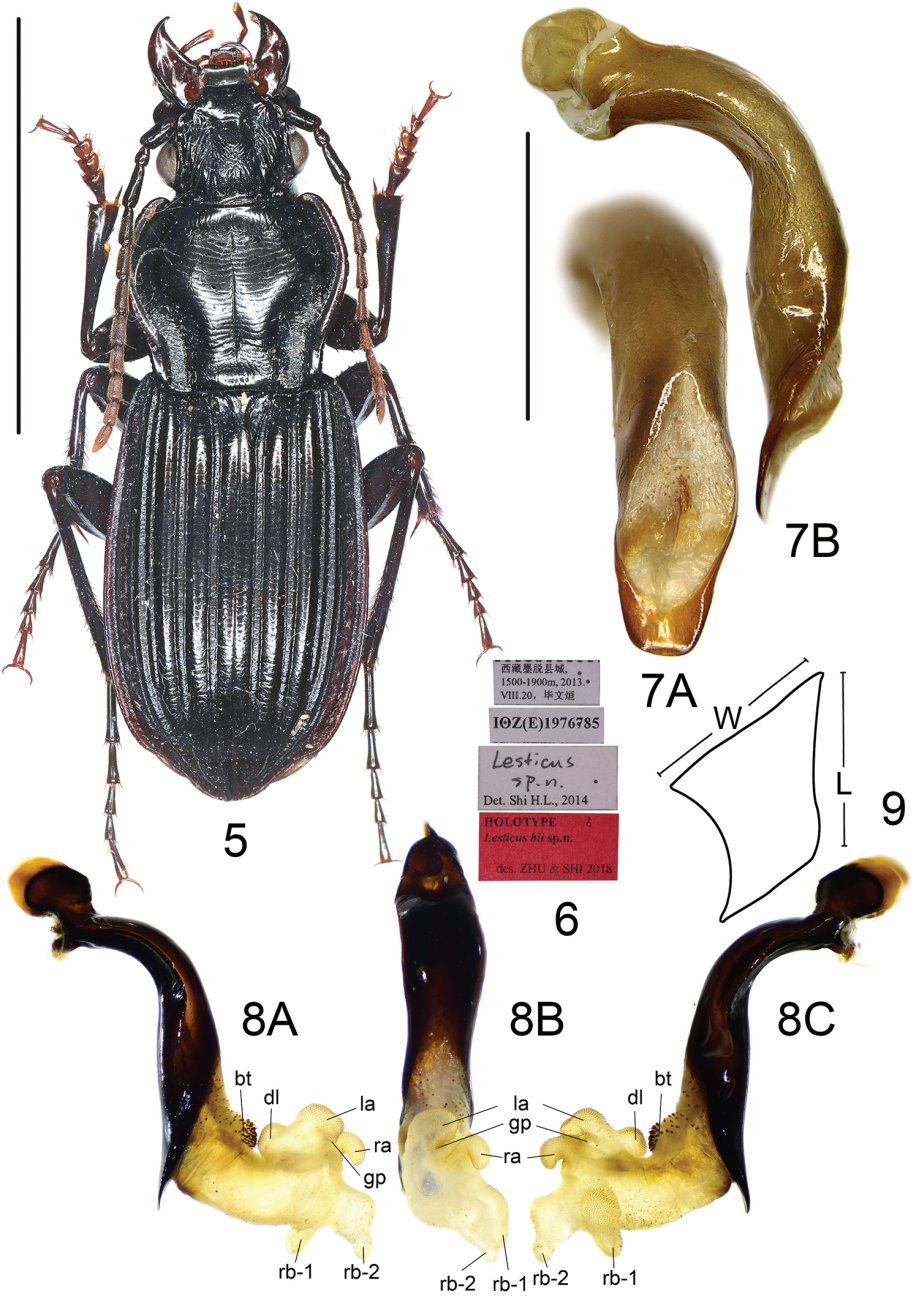
*Lesticusbii* sp. n. **5** habitus of holotype (male, Xizang, IZAS), scale bar: 10.0 mm **6** labels of holotype **7** median lobe of aedeagus (holotype) **A** dorsal view **B** left-lateral view, scale bar: 2.0 mm **8** endophallus (paratype) **A** left lateral view **B** dorsal view **C** right lateral view **9** metaepisternum.

##### Diagnosis.

Odd intervals prominently raised but not carinate, much wider than depressed even ones; third interval without setigerous pore; pronotal lateral margins crenulate through full length; basal fovea very deep; metepisternum short and wide, length subequal to its basal width; apical lamella of aedeagus truncated, shorter than basal width, without hook.

##### Comparisons.

This new species can be readily distinguished from all the other Chinese species of the genus by the prominently raised odd elytral intervals. There are three other species (*L.tricostatus* Chaudoir, *L.wittmeri* Morvan, and *L.cupricollis* Pouillaude) from the Himalayan region with similar elytral characters, but they are different from the new species by: pronotal lateral margins not crenulated; odd intervals carinate, of about the same width as the even ones; apical lamella hooked or much longer than its basal width. Among these three, the new species is most similar to *L.wittmeri* from Bhutan for they both have pronotal basal fovea markedly prolonged anteriorly, elytral first interval not carinate, and apical lamella unhooked.

##### Description.

Body length 17.8–20.0 mm, elytra’s greatest width 6.2–7.3 mm, both sexes with similar body form. Head, pronotum, elytra and appendages black, polished but without metallic lustre; basal antennomere, maxillae, labial and maxillary palpi, lateral sides of labrum, pro- and mesotarsomeres reddish brown; apical half of terminal labial and maxillary palpomeres yellow; ventral side black. Head, pronotum and elytra with isodiametric microsculpture and minute punctures.

Head with dense and fine punctures on vertex and occiput; vertex densely rugose, deeper and longitudinal near eyes, shallower and reticular at middle; labrum with anterior margin nearly straight; temporae slightly tumid behind eyes; antennal apex reaching elytra basal sixth.

Pronotum wider than head, PW/HW = 1.38–1.44; width a little greater than median length, PW/PL = 1.13–1.21; pronotum widest near anterior third; lateral margins crenulate along full length, curved near middle, distinctly sinuate before posterior angles; posterior angles rectangular, not pointed outwards; posterior margin a little narrower than anterior margin, barely extended backward on each side, middle portion gradually concave; median line fine but a little deep, not reaching posterior margin; disc with dense transverse wrinkles alongside median line; basal fovea strongly incised, inner and outer grooves indistinct, completely fused together forming large and deep depression, extending forward beyond midpoint of pronotum, and gradually fused to widened lateral channel; basal fovea finely and densely punctated and rugose, wrinkles present on middle region of pronotal base between basal fovea.

Elytra oviform, EL/EW = 1.52–1.66, gradually widened to apex, widest near posterior third; basal ridge complete, forming indistinct obtuse angle with lateral margin, humeral teeth not pointed. Intervals with shallow transverse wrinkles; odd intervals strongly raised but not carinate, much more convex and wider, about twice width of the even ones; third interval without setigerous pore; striae deeply incised, with fine and sparse punctures alongside; scutellar stria very short, apex free; parascutellar pore on base of second interval, sometimes one additional parascutellar pore present; umbilicular series on ninth interval composed of 15–20 pores almost evenly arranged. Hind wings very small.

Ventral side: propleuron and mesopleuron with dense, fine punctures; metepisternum short and wide, length a little less than its basal width (Fig. 7), with dense, fine punctures; abdominal sterna glabrous medially, finely rugose laterally, lateral sides of sternum II and sometimes sternum III as well, finely and sparsely punctate.

Legs: basal three meso- and metatarsomeres prominently carinate along full length of outer surface, fourth tarsomeres barely carinate only near base; fifth tarsomeres with 3–4 pairs of spines ventrally.

Male genitalia: median lobe of aedeagus with apical orifice opened dorsally; in lateral view, ventral margin distinctly expanded in middle, apical portion straight, turned neither ventrally nor dorsally, basal portion a little narrowed; in dorsal view, apical lamella short, length about half basal width, apex truncated; apical portion straight, oriented to neither left nor right. Endophallus (Fig. 8) short and straight, extending to dorsum, the inscribed angle between axes of aedeagus and endophallus about 90°; main portion of endophallus on dorsal side of aedeagus when everted; gonopore (**gp**, gonopore lobe folded in Fig. 8) located at a little before apical lamella, oriented to dorsal side of aedeagus. Basal tubercle (**bt**) much larger than in other species, very densely and heavily spined; basal band (**bb**) absent. Five distinct lobes recognized: dorsal lobe (**dl**) small, nearly rounded, almost touching **bt**; right basal lobe (**rb**) divided into two separate lobes; right basal lobe I (**rb-1**) elongated, bent to dorsal direction of endophallus, placed at right-dorsal surface, decorated with dense and fine scales; right basal lobe II (**rb-2**) close to **rl-1**, bent to apical direction of endophallus, apex pointing out to dorsal direction, without scales; right apical lobe (**ra**) small, on right side of **gp**, bent to dorsal direction of endophallus, apex curved; left basal lobe (**lb**) absent; left apical lobe (**la**) close to **dl**, on left side of **gp**, bent to apical direction of endophallus, apex a little bifid.

##### Distribution.

**Map 2.**
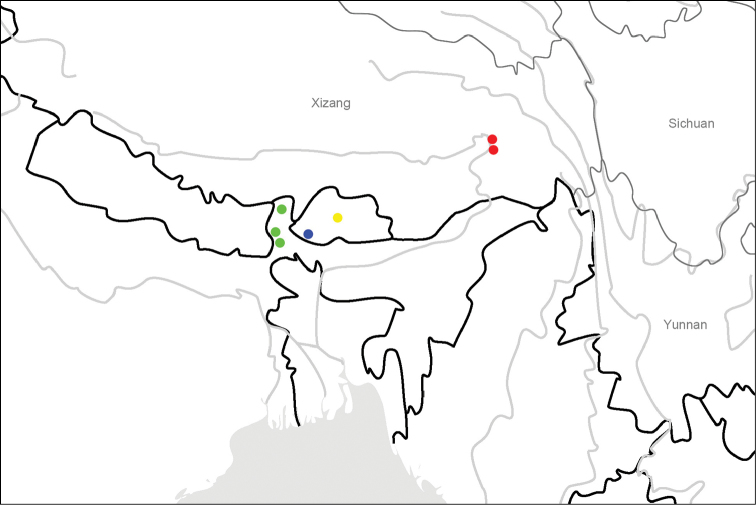
Distribution map for *L.bii* sp. n. and allied species: *L.bii* sp. n. (red); *L.tricostatus* Chaudoir (green); *L.wittmeri* Morvan (blue); *L.cupricollis* Pouillaude (yellow).

This species is known only in a few localities of Xizang, Mêdog (Map 2).

##### Etymology.

The new species is named for our friend Mr. BI Wenxuan, an excellent beetle collector, who was first to collect this rare and peculiar new species.

##### Affinities.

*Lesticusbii* sp. n. was presumed to be close to *L.tricostatus*, *L.wittmeri*, and *L.cupricollis* for the following similarities: elytral odd intervals prominently raised; third interval without setigerous pore; pronotal lateral margins distinctly sinuate before posterior angles; and all from the Himalayan region. Among them, the new species has more plesiomorphies than the others, such as: all odd elytral intervals not carinate, apical lamella unhooked. Moreover, *L.bii* sp. n. was considered to be associated with two other Himalayan species (*L.harmandi* Tschitschérine, and *L.holzschuhi* Straneo) for their similarities in: pronotal lateral margins crenulate and distinctly sinuate before posterior angles; large, deep basal fovea; elytral third interval without setigerous pore; short metepisternum. Another species from Northern Myanmar, *L.nigroviolaceus* Dubault, Lassalle & Roux was similar to the new species in external and male genitalia characters, although pronotal lateral margins are not crenulate and odd intervals not raised.. Thus, all seven species are assumed to be associated with and forming one species group defined by: (1) short metepisternum, length less than or subequal to its basal width; (2) elytra third interval without setigerous pore; (3) pronotal lateral margins distinctly sinuate before posterior angles, large, deep basal fovea; (4) relatively small body size, generally less than 24 mm.

#### Lesticus violaceous sp. n.

Taxon classificationAnimaliaColeopteraCarabidae

http://zoobank.org/E7E29088-E0F3-4699-9486-4058A21CFB73

Figs 10–13

##### Type locality.

China, Yunnan: Yingjiang, Nabang (24.71°N, 97.58°E), altitude 473 m.

##### Type material.

**Holotype**: male (IZAS), body length = 25.1 mm, pin mounted, genitalia preserved in 100% ethanol in a microvial pinned under specimen, “China, Yunnan, Yingjiang / Nabang power station, 473m / 2016-V-29, light trap, 16Y / Yang Xiaodong Leg. CCCC”; “HOLOTYPE ♂ / *Lesticusviolaceous* sp. n. / des. ZHU & SHI 2018” [red label].

**Figures 10–13.**
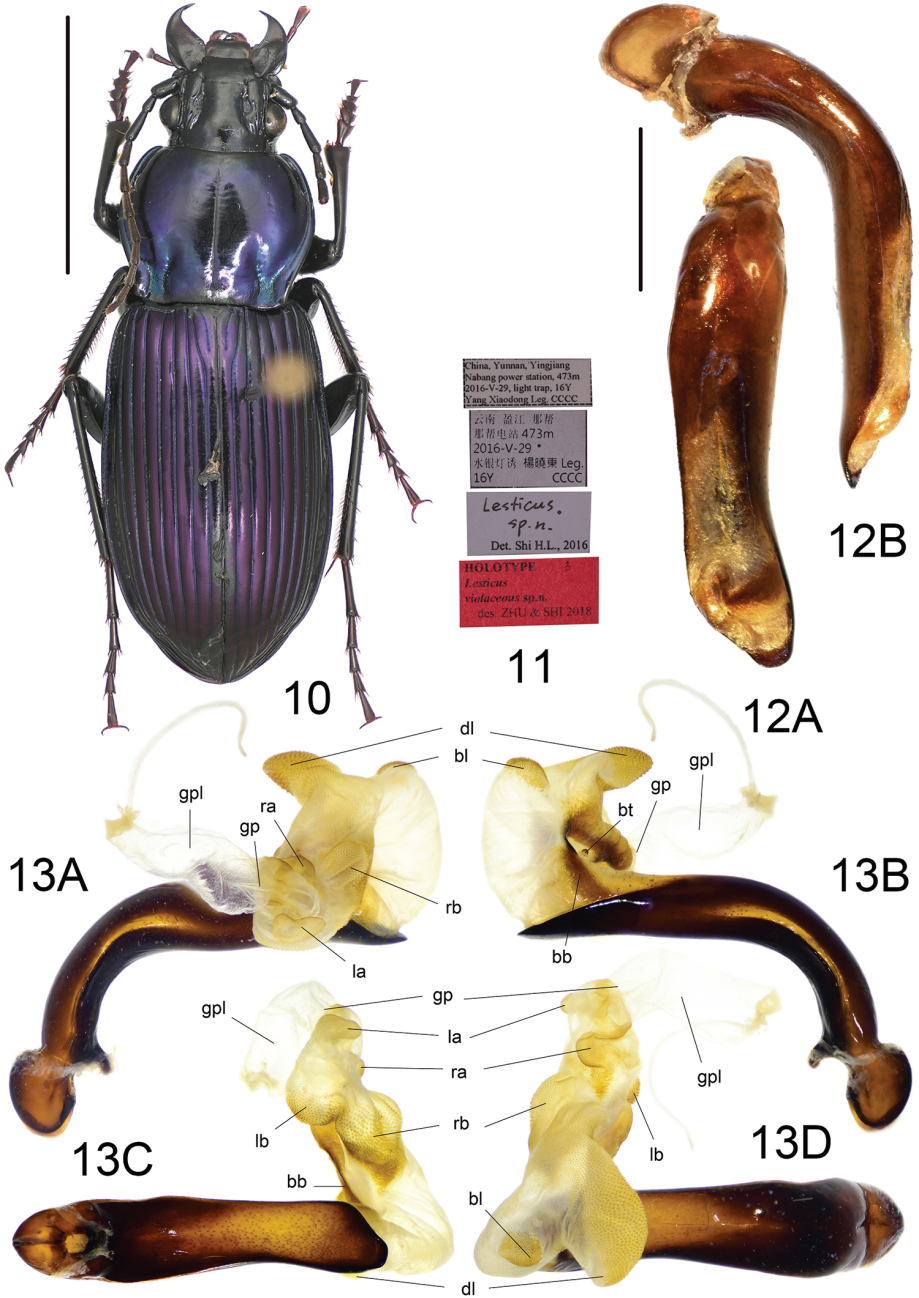
*Lesticusviolaceous* sp. n. **10** habitus of holotype (male, Yunnan, IZAS), scale bar: 10.0 mm **11** labels of holotype **12** median lobe of aedeagus (holotype) **A** dorsal view **B** left-lateral view, scale bar: 2.0 mm **13** endophallus (holotype) **A** left lateral view **B** right lateral view **C** ventral view **D** dorsal view.

##### Diagnosis.

Pronotum metallic bluish violet, elytra completely violet; pronotum lateral margins slightly sinuate before posterior angles; pronotal basal fovea deep and glabrous, with very faint wrinkles; long, narrow metepisternum, length much greater than its basal width (L/W = 1.78), distinctly punctated; median lobe of aedeagus not expanded ventrally, apex markedly deflexed to right, very short apical lamella, apex a little truncated.

##### Comparisons.

Among all *Lesticus* species from China, this new species is the only one with similar distinctly metallic color on the pronotum and elytra. From the slightly sinuate pronotal lateral margins and glabrous basal fovea, the new species is somewhat similar to *L.tristis* and *L.chalcothorax*. Besides their different color and body size, the two other species also differ from the new species in: median lobe of aedeagus distinctly expanded ventrally, apex of apical lamella more rounded.

The new species is also similar to *L.desgodinsi* from N. India and *L.episcopalis* from N. Myanmar in having a violet color on the elytra and pronotum as well as the pronotal lateral margins being somewhat sinuate before the posterior angles. Compared with the latter two species, *L.violaceous* sp. n. has less sinuate pronotal lateral margins; pronotal basal fovea less punctate; and ventral margin of aedeagus straight, not expanded near middle.

##### Description.

Body length 25.1 mm, elytra’s greatest width 9.2 mm. Head black, pronotum and elytra violet, with strong metallic lustre, pronotum somewhat blue in basal fovea and lateral channel; appendages black; tarsomeres, apical antennomeres, palpomeres and lateral sides of labrum reddish brown; ventral side black, with slightly metallic violet lustre. Head and pronotum with isodiametric microsculpture and minute punctures; elytra with isodiametric microsculpture.

Head glabrous, without coarse puncture and wrinkle; frontal impressions deep, with a few fine punctures inside; anterior margin of labrum slightly emarginate; temporae not tumid behind eyes; antennal apex reaching elytra basal tenth.

Pronotum much wider than head, PW/HW = 1.54, slightly transverse, PW/PL = 1.31, widest near middle. Lateral margins not crenulate; evenly curved at anterior two-thirds, slightly sinuate before posterior angles; posterior angles obtusely angulate, not pointed outwards; posterior margin a little narrower than anterior margin, extended slightly backward on each side. Median line deep, not reaching posterior margin; disc glabrous, without wrinkles. Basal fovea deep and narrow, inner groove nearly straight, about same length as outer one which is strongly curved, region between them deeply depressed; basal foveal area nearly glabrous, with a few very fine punctures and shallow wrinkles.

Elytra oviform, EL/EW = 1.65, gradually widened to apex, widest near posterior third; basal ridge complete, forming an indistinct obtuse angle with elytral lateral margin, humeral teeth not pointed. Intervals barely convex; striae deeply incised, with fine, sparse punctures alongside; scutellar stria long, apex free; parascutellar pore present on base of first stria; third interval with three setigerous pores: first one close to third stria, the other two close to second; umbilicular series on ninth interval composed of approximately 25 pores, sparse in middle and dense in anterior and posterior areas. Hind wings well developed.

Ventral side: propleuron with sparse, coarse punctures, a little denser on mesopleuron; long, narrow metepisternum, length much greater than its basal width (L/W = 1.78), with sparse, coarse punctures; abdominal sterna glabrous, almost impunctate, with only very shallow wrinkles on lateral sides.

Legs: basal two meso- and metatarsomeres with distinct carina only near base; fifth tarsomeres with 3–4 pairs of spines ventrally.

Male genitalia: median lobe of aedeagus with apical orifice opened dorsally; in lateral view, ventral margin straight, not expanded in middle, apical portion slightly turned dorsally before apical lamella, basal portion slightly narrowed; in dorsal view, aedeagus narrow, apical lamella very short, length approximately one-third of basal width, apex a bit truncated, apical fourth distinctly oriented to left side. Endophallus (Fig. 13) extending to dorsal-left, major portion of endophallus on left-dorsal side of aedeagus when everted; gonopore (**gp**) located at well before apical lamella, oriented to aedeagal base; gonopore lobe (**gpl**) long, a little spiral. Basal tubercle (**bt**) and basal band (**bb**) typical of the genus. Six distinct lobes recognized: dorsal lobe (**dl**) very large and compressed, on dorsal-right surface, pointing to base of aedeagus; one additional basal lobe (**bl**) present on basal-ventral side of **dl**, small and rounded, apex decorated with fine scales; right basal lobe (**rb**) large and wide, extended to ventral side of endophallus, surface with longitudinal impression; right apical lobe (**ra**) smaller than **rb**, rounded, at right-apical side of **rb**, at right surface of endophallus and close to **gp**; left basal lobe (**lb**) a little larger than **rb**, rounded, at ventral-apical side of **rb**, at ventral surface of endophallus; left apical lobe (**la**) small and compressed, close to left side **gp**, apex a little dilated and bifid.

##### Distribution.

**Map 3.**
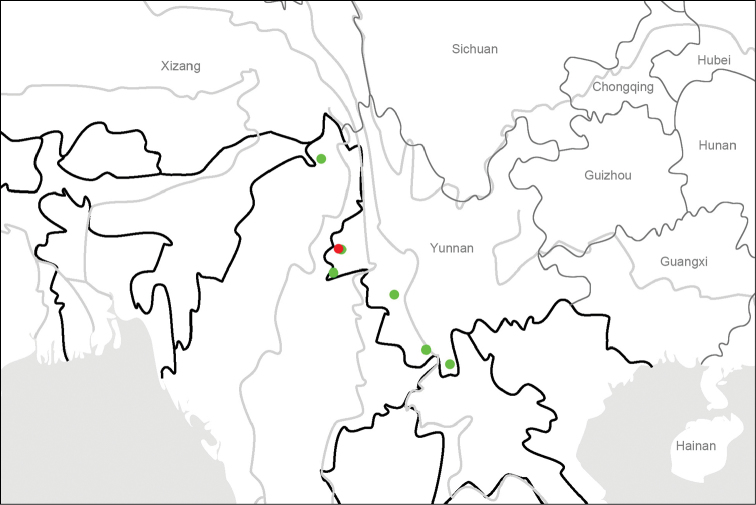
Distribution map for *L.violaceous* sp. n. and allied species: *L.violaceous* sp. n. (red); *L.rotundatus* Roux & Shi (green).

This species is known only by the holotype which was collected from Yunnan, Yingjiang, Nabang (Map 3).

##### Etymology.

The scientific name “*violaceous*” comes from Latin, referring to the violet coloration of this new species.

##### Affinities.

Among all Chinese *Lesticus* with the endophallus known, only *L.rotundatus* has similar male endophallic characters to the new species: endophallus strongly deflexed to left-dorsal side of aedeagus; gonopore pointed to the aedeagal base. Thus, a close relationship of these two species is possible, although they have quite different external and aedeagal characters.

#### Lesticus xiaodongi sp. n.

Taxon classificationAnimaliaColeopteraCarabidae

http://zoobank.org/A0022926-35D1-45F0-B9DF-8DF9A490719E

Figs 14–17

##### Type locality.

China, Yunnan: Yingjiang, Nabang (24.71°N, 97.58°E), altitude 473 m.

##### Type material.

**Holotype**: male (IZAS), body length = 27.0 mm, pin mounted, genitalia preserved in 100% ethanol in a microvial pinned under specimen, “China, Yunnan, Yingjiang / Nabang power station, 473m / 2016-V-27, light trap, 16Y / Yang Xiaodong Leg. CCCC”; “HOLOTYPE ♂ / *Lesticusxiaodongi* sp. n. / des. ZHU & SHI 2018” [red label].

**Figures 14–17.**
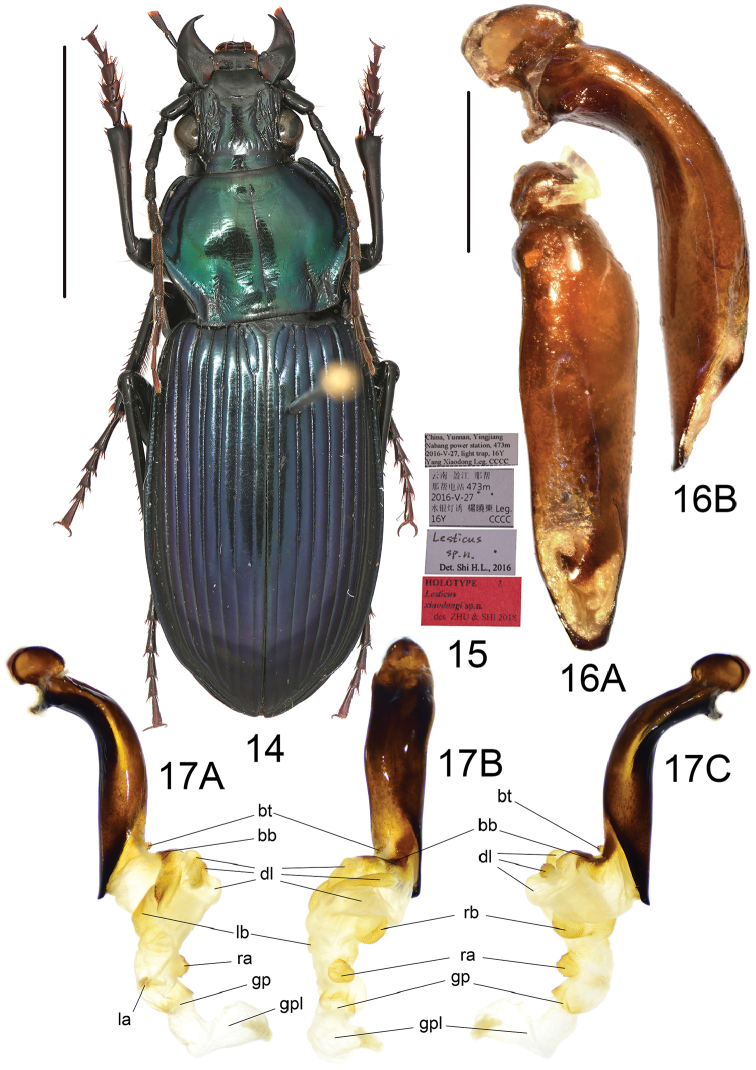
*Lesticusxiaodongi* sp. n. **14** habitus of holotype (male, Yunnan, IZAS), scale bar: 10.0 mm **15** labels of holotype **16** median lobe of aedeagus (holotype) **A** dorsal view **B** left-lateral view, scale bar: 2.0 mm **17** endophallus (holotype) **A** left lateral view **B** dorsal view **C** right lateral view.

##### Diagnosis.

Dorsal side bicolor: head and pronotum metallic bluish green, elytra dark metallic blue; pronotum lateral margins strongly sinuate before posterior angles; pronotal basal fovea deep, with fine punctures and wrinkles; posterior angles a little pointed; metepisternum long and narrow, length greater than its basal width (L/W = 1.35); median lobe of aedeagus slightly expanded ventrally, apical lamella very short, slightly thickened.

##### Comparisons.

The new species can be readily distinguished from all the other known species from China by pronotal lateral margins strongly sinuate near base. From the coloration, shape of pronotum and median lobe of aedeagus slightly expanded ventrally, this new species is most similar to *L.waterhousei* Chaudoir from N.E. India and *L.peguensis* Bates from S. Myanmar. *L.waterhousei* differs in: much larger size (33–36 mm), pronotal basal fovea coarsely rugose, and apical lamella of aedeagus much longer. *Lesticuspeguensis* differs in: pronotal posterior angles not pointed at all; apical lamella a little longer and not thickened.

##### Description.

Body length 27.0mm, elytra’s greatest width 9.7 mm. Dorsal side bicolor, head and pronotum metallic bluish green, elytra dark metallic blue; mouth part, clypeus, and appendages black; tarsomeres, apical antennomeres, terminal palpomeres reddish brown; ventral side black, without metallic lustre. Head, pronotum and elytra with isodiametric microsculpture and minute punctures.

Head: vertex nearly glabrous, with very fine shallow wrinkles; frontal impressions deep; coarse longitudinal wrinkles along inner margins of eyes; anterior margin of labrum distinctly emarginate; temporae not tumid behind eyes; antennal apex reaching elytra basal sixth.

Pronotum much wider than head, PW/HW = 1.52, slightly transverse, PW/PL = 1.32; widest near middle. Lateral margins not crenulate, evenly curved in middle, strongly sinuate before posterior angles; posterior angles nearly rectangular, pointed a little outward; posterior margin a little narrower than anterior, extended backward on each side. median line is deep, not reaching posterior margin; disc with very shallow transverse wrinkles alongside median line. Basal fovea deep, inner groove straight, a little longer than curved outer groove, region between them deeply depressed; basal foveal area with some fine but distinct punctures and wrinkles.

Elytra oviform, EL/EW = 1.65, gradually widened to apex, widest near posterior third; basal ridge complete, forming an indistinct obtuse angle with elytral lateral margin, humeral teeth not pointed. Intervals barely convex, striae deeply incised, with fine and dense punctures alongside; scutellar stria moderately long, apex conjoined to first stria; parascutellar pore located at base of first stria; third interval with three setigerous pores: first one close to third stria, other two close to second; umbilicular series on ninth interval composed of approximately 25 pores, sparse in middle and dense in anterior and posterior areas. Hind wings well developed.

Ventral side: propleuron and mesopleuron with dense, coarse punctures; metepisternum long and narrow, length greater than its basal width (L/W = 1.35), with sparse, coarse punctures; abdominal sterna glabrous in middle, with dense, coarse punctures on lateral sides of sternum II and sternum III, and shallow wrinkles on lateral sides of all sterna.

Legs: three basal metatarsomeres with distinct carina along almost the full length of outer surface, three basal mesotarsomeres, and fourth with weaker carina only near base; fifth tarsomeres with 3–4 pairs of spines ventrally.

Male genitalia: median lobe of aedeagus with apical orifice opened dorsally; in lateral view, ventral margin slightly expanded in middle, basal portion not narrowed, apical lamella slightly thickened and turned ventrally; in dorsal view, aedeagus gradually narrows from middle to apex, apical lamella very short, length approximately one-third of basal width, apex a little truncated; apical portion a little inclined to right side. Endophallus (Fig. 17) straight, extending to apex, the included angle between axes of aedeagus and endophallus about 20°; major portion of endophallus located beyond apical lamella, inclined a little to left; gonopore (**gp**) located at well beyond apical lamella, oriented to aedeagal apex; gonopore lobe (**gpl**) long, a little spiral. Basal tubercle (**bt**) typical of the genus; basal band (**bb**) short, obsolete at apex, not reaching left surface of **dl**. Five distinct lobes recognized: large dorsal lobe (**dl**), divided into three sub-lobes by longish grooves: basal one longitudinal; middle one on right side of basal one, very narrow and transverse; apical one transverse, larger than the other two, without scales; single right basal lobe (**rb**), large and rounded, on right-apical side of **dl**, decorated with dense scales; right apical lobe (**ra**) smaller than **rb**, rounded, close to **gp**; left basal lobe (**lb**) very large and flat, near apex of **bb**; left apical lobe (**la**) very small, nearly imperceptible.

##### Distribution.

**Map 4.**
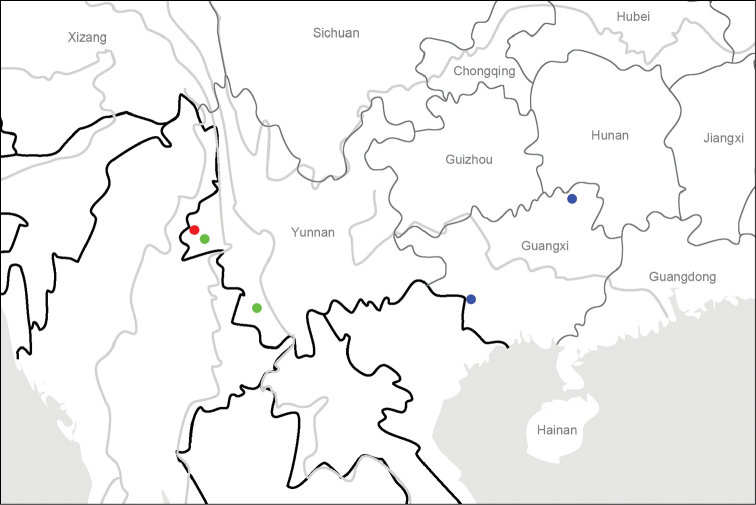
Distribution map for *L.xiaodongi* sp. n. and allied species: *L.xiaodongi* sp. n. (red); *L.tristis* Roux & Shi (green); *L.solidus* Roux & Shi (blue)

This species is known only by the holotype, which was collected from Yunnan, Yingjiang, Nabang, the same locality as the previous new species (Map 4).

##### Etymology.

The new species is named for our friend Mr. Yang Xiaodong, who collected the holotype of this beautiful and rare new species.

##### Affinities.

Among all Chinese *Lesticus* we studied, *L.tristis* and *L.solidus* have the most similar male endophallic characters to the new species: endophallus straight, major portion extending to apical direction of aedeagus; in lateral view, the angle between axes of endophallus and aedeagus less than 30°. Moreover, these three species all have pronotal basal fovea not well punctated, and metepisterna much longer than its basal width. This suggests a close relationship among the three species.

#### Lesticus solidus

Taxon classificationAnimaliaColeopteraCarabidae

Roux & Shi, 2011

Figs 18–23

Lesticus solidus Roux & Shi, 2011: 94 (type locality: Maoershan, Guangxi, holotype in IZAS).

##### Material examined.

**Holotype of *L.solidus***: female (IZAS), body length = 29.1 mm, pin mounted, “Gaozhai, Maoershan Mt., / Xing’an, Guangxi, CHINA / 900m, by light trap, / 3.VIII.2005”, “IOZ(E) 1891818”, “HOLOTYPE♀ / *Lesticussolidus* / Roux & Shi 2011 / Des. Roux &Shi, 2011” [red label]; one male (IZAS), “Guangxi, Xing’an, Yong’an, 2009.VII.13 5-2”, “IOZ(E) 1976781”; one female (IZAS), “China: Guangxi Prov, Chongzuo, Daxin county, Shuolong town, Heishuihe; 22.8194N, 106.8639E, 345m”, “2016.IV.27N, on dead log; Shi H. L., Liu Y. & Liu Y. Z. lgt., Institute of Zoology., CAS.”, one female (IZAS), “Guangxi Maoershan”, “IOZ(E) 1976782”.

**Figures 18–23.**
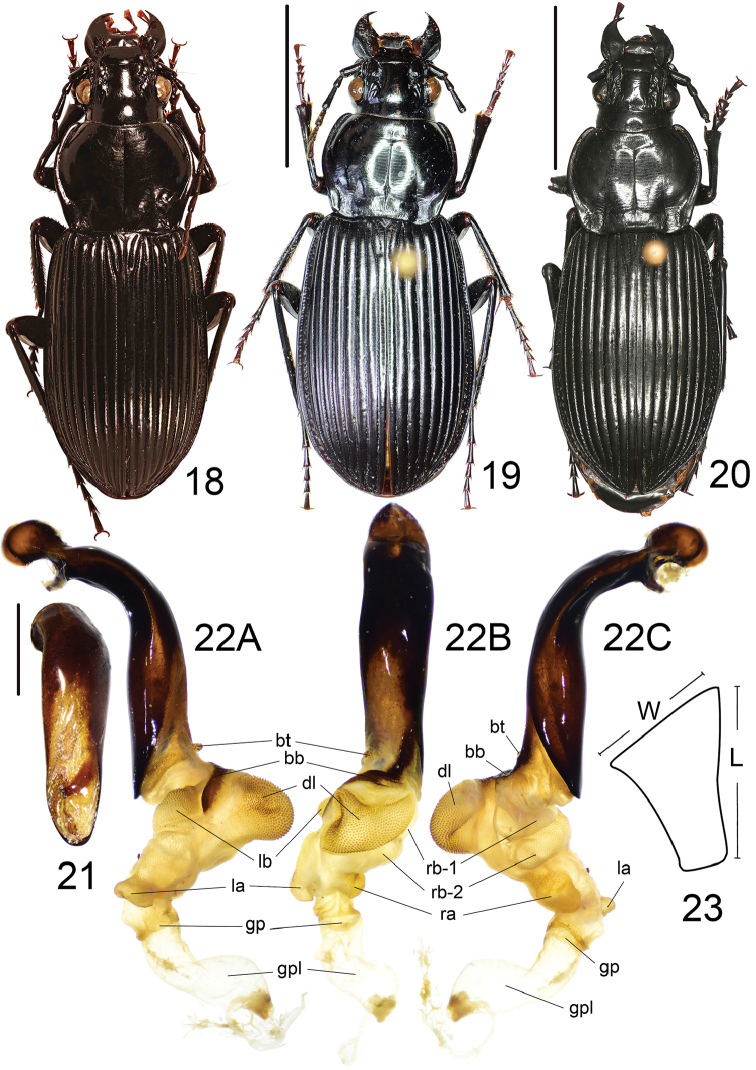
*Lesticussolidus* Roux & Shi. **18** holotype of *L.dubius* (male, Guangxi, MNHN) **19** holotype of *L.solidus* (female, Guangxi, IZAS), scale bar: 10.0 mm **20** habitus of a male from Guangxi, scale bar: 10.0 mm **21** median lobe of aedeagus, dorsal view, scale bar: 2.0 mm **22** endophallus **A** left lateral view **B** dorsal view **C** right lateral view **23** metaepisternum.

##### Notes on synonym.

*Lesticussolidus* Roux & Shi was described based on a single female from Maoershan (Guangxi, China). Two years later, *L.dubius* Dubault, Lassalle & Roux was described from exactly the same locality based on four specimens including both sexes. In the present study, we compared the female holotype of *L.solidus* (Fig. 19) and a male (Fig. 20) and found that they are perfectly in accordance with the type locality and male genitalia illustrations (Dubault et al. 2013) of *L.dubius*. They are different only in: *L.solidus* with very faint violet lustre on elytra, and elytra more widened to apex. These differences also correspond with the original description of *L.dubius*. Among all our examined materials, the females generally exhibited elytra more widened to apex. Thus we considered it as a sexual dimorphic character. About the very faint violet lustre of *L.solidus*, it is more likely an individual variation rather than a specific character. This case is similar to head and pronotum color variations of *L.dubius* which were noted by Dubault et al. (2013). So, we herein synonymize *L.dubius* with *L.solidus*.

##### Supplementary descriptions on endophallus

(Fig. 22): Endophallus straight, extending to apex, the angle formed by the axes of aedeagus and endophallus about 5°; major portion of endophallus located beyond apical lamella; gonopore (**gp**) located at a level well beyond apical lamella, pointing to aedeagal apex; gonopore lobe (**gpl**) long, a little spiral. Basal tubercle (**bt**) and basal band (**bb**) typical of the genus. Six distinct lobes recognized: dorsal lobe (**dl**) very large and rounded, on dorsal surface and sharply pointed out; right basal lobe (**rb**) divided into two separate lobes; right basal lobe I (**rb-1**) large and a little compressed, close to apical lamella, surface without scales; right basal lobe II (**rb-2**) a little larger than **rb-1**, rounded, at apical side of **rb-1**, decorated with sparse, fine scales; right apical lobe (**ra**) a little smaller than **rb-1**, rounded, between **rb-2** and **gp**; left basal lobe (**lb**) large and compressed, close to apex of **bb**; left apical lobe (**la**) small and compressed, pointing out sharply, at left side of **gp**.

## Discussion

*Lesticus* is an Oriental genus with more than a hundred described species (Roux et al. 2016), and several of them have impressive metallic color. Although this genus was well defined, different species in the genus usually have similar external and male genital characters, making species determination generally difficult. Moreover, due to the limited number of available characters for systematic study, solving species relationships in *Lesticus* was never attempted before. In Pterostichini, the endophallic characters were of value for species relationships (Shi and Liang 2015). Thus, in an attempt to resolve part of the species relationships in *Lesticus*, we studied the endophallus of all available Chinese species (14 of 18 known species). Based on the comparative morphology study, preliminary conclusions on endophallic character evolution and phylogenetic considerations within *Lesticus* are presented below.

### Endophallus categories in *Lesticus*

According to the materials and endophallus illustrations provided by Roux et al. (2016) that we examined, the endophallus shape is highly variable in *Lesticus*, but there are some regularities revealed. We classified here the endophallus of species studied (14 Chinese species and 19 species from other faunas) into four types, mainly based on the orientation of the gonopore and rotation or deflection of the endophallic axis.

### Type I

The endophallus type I (as shown in Figs 8, 17, 22, 25 and 27) is the most common type among all examined species, and all other types can be explained as modifications based on this type. The enodphallus type I has the following character states:

Endophallic axis nearly straight; extending to genital apex, or more or less deflected to dorsum, with the angle inscribed between axes of aedeagus and endophallus (**AE-angle**, axis of endophallus was the line between midpoint of apical orifice and gonopore) between 5° and 80°; gonopore oriented to endophallic apex; major portion of endophallus located at apical, apical-dorsal or dorsal side of aedeagus. Seven groups of features recognized: (1) basal tubercle (**bt**) at the base of endophallus, generally very small, its surface with very dense scales, **bt** rarely disappearing; (2) basal band (**bb**), a long and narrow chitinized band, beginning at right margin of apical orifice, then surrounding base of **dl**, and ending at left surface of it, **bb** sometimes shortened or obsolete; (3) dorsal lobe (**dl**) on dorsal basal surface of endophallus, close to dorsal margin of aedeagus, generally large and rounded, usually the largest lobe on endophallus and decorated with very coarse scales, sometimes divided into sub-lobes or separate lobes; (4) right basal lobe (**rb**) on right basal surface of endophallus, usually divided into two separate lobes; (5) right apical lobe (**ra**) on right surface of endophallus, close to gonopore, generally much smaller than **rb**; (6) left basal lobe (**lb**) on right basal surface of endophallus, sometimes divided into two separate lobes or almost imperceptible; (7) left apical lobe (**la**) on left surface of endophallus, close to gonopore, generally much smaller than **lb**. All lobes generally decorated with scales at least on apex.

A total of 10 Chinese species have the type I endophallus: *L.solidus* Roux & Shi, *L.tristis* Roux & Shi, *L.xiaodongi* sp. n., *L.chalcothorax* (Chaudoir), *L.sauteri* Kuntzan, *L.perniger* Roux & Shi, *L.bii* sp. n., *L.deuvei* Dubault & Roux, *L.taiwanicus* Roux & Shi, *L.auricollis* Tschitschérine. Among these 10, two different forms (or sub-types) were recognized: (1) the **chalcothorax-form** (Figs 17, 23): endophallus almost straight, only slightly or not deflexed to dorsum, AE-angle 5° to 55°; gonopore at apical direction of aedeagus; **dl** very large, placed on dorsal surface; **dl** and **rb** sometimes divided; without additional basal lobe. Five Chinese species have the chalcothorax-form: *L.solidus*, *L.tristis*, *L.xiaodongi*, *L.chalcothorax*, and *L.sauteri*; (2) the **auricollis-form** (Fig. 27): endophallus markedly deflexed to dorsum, AE-angle 70° to 80°; gonopore at dorsal direction of aedeagus; **dl** placed on dorsal-right surface; **dl**, **rb** and **lb** all divided into two separate lobes; **bb** short and wide; one additional basal lobe (**bl**) present at right basal side of **dl**. Three Chinese species have the auricollis-form: *L.deuvei*, *L.taiwanicus* and *L.auricollis*.

The remaining two species, *L.perniger* (Fig. 25) and *L.bii* (Fig. 8), are special within type I due to: **bt** less pointed than other species; **lb** completely absent; **rb** located well before midpoint of endophallus. But some other specialized differences might contradict their affinities: **bt** flat but very large and coarsely spined in *L.bii*; **bb** absent in *L.bii*, very long, reaching midpoint of endophallus in *L.perniger*; **la** with a heavily chitinized piece in *L.perniger*; **rb** divided into two separate lobes in *L.bii*.

**Figures 24–28.**
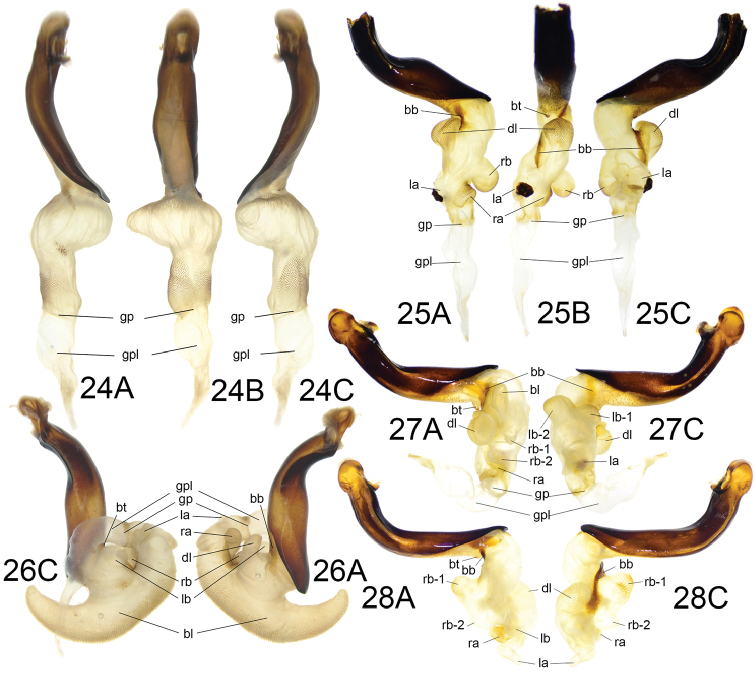
Endophallus of species representatives. **A** left lateral view **B** dorsal view **C** right lateral view **24***Trigonotomalewisi* Bates from Yunnan (type I) **25***Lesticusperniger* Roux & Shi from Yunnan (type I) **26***Lesticusmagnus* (Motschulsky) from Liaoning (type III) **27***Lesticusauricollis* Tschitschérine from Fujian (type I) **28***Lesticusinsignis* Gestro from Sabah (type IV).

Based on the endophallus illustrations provided by Roux et al. (2016), 11 species of the Chinese fauna also have an endophallus of type I. Four of them have the auricollis-form endophallus: *L.andamanensis* (Chaudoir), *L.mouhoti* (Chaudoir), *L.nubilus* Tschitschérine and *L.waterhousei* (Chaudoir); and another four have the chalcothorax-form: *L.buqueti* (Castelnau), *L.indus* (Tschitschérine), *L.kangeanensis* Dubault et al. and *L.stefanschoedli* Kirschenhofer. The remaining three species cannot be categorized as either form: *L.tricostatus* Chaudoir, *L.cupricollis* Pouillaude and *L.desgodinsi* Tschitschérine. However, the former two species are clearly similar to *L.bii* sp. n. for both external and endophallic similarities. Besides *Lesticus*, some species of *Trigonotoma* (such as *T.lewisi* in Fig. 24) also have the endophallus straight and the gonopore placed well beyond the aedeagus apex (similar to the chalcothorax-form), but in *Trigonotoma* the endophallus generally has fewer or no lobes.

### Type II

The endophallus type II (Figs 4, 13) with three Chinese species representatives (*L.auripennis* sp. n., *L.violaceous* sp. n. and *L.rotundatus* Roux & Shi) is characterized by: endophallic axis markedly deflexed to left, gonopore oriented to left-basal side of aedeagus; **dl** placed on right side of endophallus; one additional basal lobe (**bl**) present on basal-ventral side of dl. Among these three, *L.auripennis* is different from the other two species by: **bl** located on right surface, almost reaching midpoint of endophallus; gonopore oriented to left-apical side of aedeagus. Another species, *L.assamicus* (Kuntzen) from north India, is also known to have the type II endophallus.

Type II endophalli are well characterized by the endophallic axis markedly deflexed to left, but it seems that such a character cannot support close relationships among species with the type II endophallus. Based on other similarities among related species, we inferred that the type II endophallus might be derived from type I by the elongation of the endophallic base and deflection of the endophallic axis to the left, with *L.violaceous* and *L.rotundatus* derived from the auricollis-form, and *L.auripennis* probably from a *L.perniger*-like form.

### Type III

The endophallus type III (Fig. 26) is a peculiar type only found in *L.magnus* (Motschulsky) which has a wide distribution in East Asia. It is characterized by: endophallic axis short and markedly deflexed to aedeagal base; a large conical basal lobe (**bl**) forming conspicuous ventral projection; main portion of endophallus located on dorsal side of aedeagus; gonopore oriented to ventral side. Although peculiar, type III was assumed to have transformed from the auricollis-form of type I by: endophallic apex more deflexed to basal-ventral side; all primary lobes reduced in size and restricted at dorsal margin of endophallus; the presence of a very large additional basal lobe (**bl**).

### Type IV

The endophallus type IV (Fig. 28) is characterized by the endophallic axis markedly helical on the basal portion. No Chinese species has a type IV endophallus; all known species (nine) of this type are from the Malay Archipelago (Philippines, Borneo, Java). The highly specialized helix-formed endophallus and its regional distribution may suggest close relationships among species of the type IV endophallus.

Although highly specialized, the type IV endophallus was still inferred as a modified type from the type I, and homologies of all lobes can be distinguished (Fig. 28). From the chalcothorax-form of type I, type IV is assumed to be transformed by: **dl** moved to apex and deflexed to left side; endophallic base before **dl** elongated and rotated; **rb-1** enlarged and protuberant; **rb-2** generally small; **lb** obsolete; **la**, **ra**, **bb**, and **bt** similar to those in type I; main portion of endophallus located on dorsal side of aedeagus; gonopore generally oriented to basal-dorsal side.

### Chinese *Lesticus* species with unknown endophallus

There are four Chinese *Lesticus* species with the endophallus unknown. Based on external and aedeagal characters, we briefly discuss here their presumed relationships and predicted endophallic types.

*Lesticusfukiensis* Jedlička and *L.wrasei* Dubault, Lassalle & Roux: These two species are very similar in habitus and aedeagus features and have adjacent distributions. Compared to other species from China, they are most similar to and presumed to be closely related to *L.auripennis* sp. n., sharing the short and wide metepisternum; aedeagus only slightly or not expanded ventrally; in dorsal view, apical portion of aedeagus very slightly oblique to right side (might be associated with left deflection of endophallus). Thus, these two species are hypothesized to have endophallus character states similar to the type II of *L.auripennis*.

*Lesticusater* Roux & Shi: Based on the short, wide metepisternum, this species was considered as belonging to the same group of *L.perniger* (see discussion under *L.auripennis*). But from the almost impunctate ventral surface and the peculiar shape of aedeagal apex, *L.ater* is an outlier in this group and its close allies are unclear. Nevertheless, from the symmetrical aedeagal apex (in dorsal view not oblique to right or left side), *L.ater* is hypothesized to have type I endophallus similar to *L.perniger*.

*Lesticuspraestans* (Chaudoir): This species is assumed to be close to *L.deuvei* based on their similarities in several external characters, for example: metepisternum longer than its basal width; pronotal with metallic color; basal fovea distinctly punctate. Thus, an auricollis-form endophallus is hypothesized.

### Evolution of other characters

It is difficult to infer species relationships in *Lesticus* from external characters only, because only very few characters are available, and many of them have lot of variation in the genus. Similar problems were also encountered when distinguishing similar species and determining identification keys. Nevertheless, there are still some character transformations that are widely presented in *Lesticus* and important in species determination. Some of the transforming polarities seem to be clear, while others are more complex. We selected here some of the most common transformed characters in *Lesticus*, inferred their transforming polarity and preliminary evaluated their taxonomic value.

**Coloration**: The metallic or black pronotum is a constant character in most species and seems to have some systematic importance. The non-metallic color could be apomorphies. The head coloration always conforms with that of the pronotum, but elytra coloration should be regarded as an independent character, although in many groups it seems to have some linkage with pronotal coloration.

**Punctures**: The punctures of pronotal basal fovea, elytra intervals, propleuron and metepisternum are useful to distinguish similar species, but they are variable in some species. Generally, punctures on different body parts are somewhat linked, but it seems that these punctate characters have very little importance when inferring species relationships.

**Pronotum shape**: The pronotum shape is variable and important in identification of *Lesticus* species. Different shapes of pronotum are recognizable, among which the sub-quadrate shape (as in *L.magnus*) is inferred to be plesiomorphic, while round (as in *L.andamanensis*) and cordiform (as in *L.janthinus*) are apomorphies. Some species relationships might be inferred by specialized pronotal shape. For instance, the strongly cordiform pronotum may suggest a close relationship of *L.janthinus* with its allies from Indonesia.

**Pronotal margin**: About 13 species with quite different external appearance have crenulate pronotal lateral margins. This apomorphic character state is useful in species identification and relationship inference, but it might have homoplastic transformations also.

**Elytral discal pores**: Most species of the genus have three setigerous pores on elytral third interval, but some of them have only one or two pores, and a very few species have none. It is clear that three pores is plesiomorphic, and the reduced number of discal pores might support some monophyletic groups.

**Elytral interval**: Seven species of *Lesticus* have elytra modified by odd intervals raised, ridged or widened. This apomorphic state occurs in two groups having rather distant distributions (Himalayan Mountains and Lesser Sunda Islands) and some other characters are also different (i.e. elytral discal pores). Thus, these two groups might not be close and homoplastic transformations are inferred.

**Metepisternum**: The length of metepisternum has important value for species identification and the definition of some species groups. It seems that this character has distinct binary states: the shortened form (associated with reduced hind wings) with its length shorter or subequal to its basal width, and the normally longer form (associated with developed hind wings) with a length more than 1.3 times its basal width. Similar to most genera of Carabidae, the shortened metepisternum is apomorphic, and associated with the reduction of hind wings and adaptation to mountain habitat. But there seem to be several parallelisms in its character transformations.

### Infra-genera taxonomy of *Lesticus*

Presently, the genus *Lesticus* is divided into three subgenera as accepted in catalogues (Lorenz 2005, Löbl and Löbl 2017). This infra-genera taxonomy comes primarily from Tschitschérine (1900) with three subgenera defined as follows: *Lesticus* s. str. (type species: *Lesticusjanthinus* Dejean) have cordiform pronotum, lateral margins strongly sinuate before posterior angles; short metepisternum, the outer margin not longer than basal width. *Celistus* Tschitschérine (type species: *Triplogeniusandamanensis* Chaudoir) have round pronotum, lateral margins evenly curved; short metepisternum, outer margin not longer than basal width. *Triplogenius* Chaudoir (type species: *Trigonotomabicolor* Laporte [= *Omaseusviridicollis* Macleay]) have variable pronotum shapes, usually near quadrate; long metepisternum, outer margin distinctly longer than basal width.

However, this subgeneric system was not accepted in many important revisions (i.e. Jedlička 1962, Roux et al. 2016), and many species described later without original subgenera assignment were subsequently placed in subgenera very casually by cataloguers. For instance, two very close species, *L.auricollis* and *L.deuvei*, were assigned to different subgenera (Löbl and Löbl 2017). Moreover, many species presently assigned to *Lesticus* s. str. do not have the typical cordiform pronotum of the subgenera, and some of them even have elongated metepisternum making the definition of subgenera unclear.

In the present study, we examined all Chinese *Lesticus* species and compared them to many foreign species according to some important morphology characters. We found that in *Lesticus* the short metepisternum is not always in accordance with cordiform or round pronotum, and neither pronotum shape nor metepisternum length can support a monophyletic group inferred by the endophallic characters (see above). Both of the type species of *Lesticus* and *Triplogenius* have similar type IV endophalli which supports these two species being actually closely related. All the above evidence, object of the present infra-general taxonomy, originated with Tschitschérine (1900).

In conclusion, except the monotypic *Celistus*, the other two subgenera in the present concept are obviously not monophyletic, but an improved infra-general system cannot be proposed at this time. Thus, we suggest that it is better not to introduce subgenera in the genus *Lesticus* before a comprehensive phylogenetic study is completed.

## Catalogue of *Lesticus* from China

***Lesticusater* Roux & Shi, 2011**

Roux and Shi 2011: 95 (Type locality: Jinping, Yunnan; Holotype in IZAS); Roux, Lassale and Dubault 2016: 356.

**Chinese common name**: 乌劫步甲 (Wū Jié Bù Jiă)

**Distribution.** Yunnan (Jinping).

***Lesticusauricollis* Tschitschérine, 1900**

Tschitschérine 1900: 174 (Type locality: Bangkok; Holotype in MNHN); Kuntzen 1911: 165; Jedlička 1962: 326; Dubault et al. 2007: 217; Roux et al. 2016: 332.

**Chinese common name**: 金胸劫步甲 (Jīn Xiōng Jié Bù Jiă)

**Distribution.** Fujian, Jiangxi, Zhejiang, Hunan, Guangdong, Guangxi, Hainan; Thailand; Vietnam.

***Lesticusauripennis* sp. n.**

**Chinese common name**: 金鞘劫步甲 (Jīn Qiào Jié Bù Jiă)

**Distribution.** Guangdong (Nanling).

***Lesticusbii* sp. n.**

**Chinese common name**: 毕氏劫步甲 (Bì Shì Jié Bù Jiă)

**Distribution.** Xizang (Mêdog).

***Lesticuschalcothorax* (Chaudoir, 1868)**

Chaudoir 1868: 153 (*Triplogenius*; Type locality: Cambodge; Lectotype in MNHN); Bates 1889a: 105 (*Triplogenius*); Kuntzen 1911: 165; Andrewes 1921: 178 (*Triplogenius*); Jedlička 1962: 327; Dubault et al. 2007: 211; Roux et al. 2016: 334.

**Synonym**: *Triplogeniusbouqueti* Laporte de Castelnau: Bates 1889b: 276.

**Synonym**: *Lesticuslakhonus* Tschitschérine, 1900: 171 (Type locality: «Lakhon» [=Nakhon Phanom, Thailand]; Holotype in MNHN); Rouxet al. 2016: 334 (synonymy designation).

**Chinese common name**: 绿胸劫步甲 (Lǜ Xiōng Jié Bù Jiă)

**Distribution.** Jiangxi, Zhejiang, Fujian, Hunan, Guangdong, Guangxi, Guizhou, Yunnan; Myanmar; Thailand; Vietnam; Cambodia.

***Lesticusdeuvei* Dubault & Roux, 2006**

Dubault and Roux 2006: 189 (Type locality: Huaping, Guangxi; Holotype in MNHN); Roux et al. 2016: 364.

**Chinese common name**: 德夫劫步甲 (Dé Fū Jié Bù Jiă)

**Distribution.** Guangxi (only known from N.E. Guangxi).

***Lesticusfukiensis* Jedlička, 1956**

Jedlička 1956: 213 (Type locality: Kuatun [=Guadun, Fujian]; Holotype in NMPC); Jedlička 1962: 323; Dubault et al. 2008: 466; Roux et al. 2016: 358.

**Chinese common name**: 福建劫步甲 (Fú Jiàn Jié Bù Jiă)

**Distribution.** Fujian (only known from N. Fujian).

***Lesticusmagnus* (Motschulsky, 1860)**

Motschulsky 1860: 5 (*Omaseus*; Type locality: Japan; Holotype in ZMUM); Heyden, 1879: 332 (Triplogenius); Andrewes 1933: 14 (*Pterostichus*); Jedlička 1962: 322 (misspelled as *maguus*); Roux et al. 2016: 362.

**Synonym**: *Omaseusingens* Morawitz, 1863: 54 (Type locality: Jesso; Syntype in ZMUM). Chaudoir 1868: 154 (*Triplogenius*; synonym designation); Bates 1873: 284 (*Triplogenius*); Kuntzen 1911: 175.

**Chinese common name**: 大劫步甲 (Dà Jié Bù Jiă)

**Distribution.** Liaoning, Beijing, Hebei, Shaanxi, Gansu, Shandong, Hubei, Hunan, Jiangsu, Anhui, Jiangxi, Shanghai, Zhejiang, Sichuan; Japan; Korea.

**Notes**: This species was recorded from Taiwan (Jedlička 1962), but no any confirmed specimen was examined hereafter. We inferred the record from Taiwan was based on the misidentification of *L.sauteri* and excluded this regional record.

***Lesticusperniger* Roux & Shi, 2011**

Roux and Shi 2011: 98 (Type locality: Lancang, Yunnan; Holotype in IZAS); Roux, Lassale and Dubault 2016: 352.

**Chinese common name**: 黑劫步甲 (Hēi Jié Bù Jiă)

**Distribution.** Yunnan; Guangxi*.

*New province record with examined materials: 1 female (SCAU), “Guangxi, Maoershan, Tongmujiang source, 1250m, 2003.8.25, TIAN M. Y.”. 1 male (SNU), “China: Guangxi Prov., Lingui County, Huaping N. R., Anjiangping, 1300m, 18-VII-2011, PENG Zhong leg.”. 1 male (IZAS), “China, Guangxi, Xingan, Jinshiguiyan, 1229m, 2016-VII-09 light trap, LU Y. Q. Leg. CCCC”. 1 male (NHMB), “China, Guangxi Reg. Miaoershan mts., 500–1200m, south slope 26.-29.vi.1997”.

***Lesticuspraestans* (Chaudoir, 1868)**

Chaudoir 1868: 154 (*Triplogenius*; Type locality: Hongkong; Lectotype in MNHN); Jedlička 1962: 323; Dubault et al. 2007: 212; Roux et al. 2016: 344.

**Chinese common name**: 立劫步甲 (Lì Jié Bù Jiă)

**Distribution.** Hongkong, Guangdong*.

*New province record with examined materials: 2 females (IZAS), “China, Guangdong, Shengzhen, Xianhu Botanical Garden, 22.58184, 114.16440 34m, 2014.5.3 N, Liang H. B., Huang X. L., Institute of Zoolgy, CAS”.

***Lesticusrotundatus* Roux & Shi, 2011**

Roux and Shi 2011: 102 (Type locality: Shuangjiang, Yunnan; Holotype in IZAS); Roux et al. 2016: 366.

**Chinese common name**: 圆胸劫步甲 (Yuán Xiōng Jié Bù Jiă)

**Distribution.** Yunnan (Shuangjiang, Ruili, Xishuangbanna*, Yingjiang*), Myanmar* (Putao).

*New record with examined materials: 1 female (IZAS), “Guomenshan, Nabanhe, Yunnan Prov. alt. 1100m, 23-VII-2005, LI & LI leg.”. 1 female (CCCC), “13Y2013-X-12, Yunnan, Jinghong, Menglong 622m, rubber forest YANG X. D. Leg.”. 1 female (CCCC), “China, Yunnan, Yingjiang, Nabang power station, 473m, 2016-V-29, light trap, 16Y, Yang Xiaodong Leg. CCCC”. 1 male (IZAS), “Myanmar, Kachin State, Putao county, Ba aye, way to Nahteukhu. 27.3183, 97.3894, 2016.12.8 576m”.

***Lesticussauteri* Kuntzen, 1911**

Kuntzen 1911: 175 (Type locality: Taiwan; Syntypes in MNHU); Jedlička 1962: 322; Dubault et al. 2008: 465; Roux et al. 2016: 360.

**Chinese common name**: 绍氏劫步甲 (Shào Shì Jié Bù Jiă)

**Distribution.** Taiwan.

***Lesticussolidus* Roux & Shi, 2011**

Roux and Shi 2011: 105 (Type locality: Xing’an, Guangxi; Holotype in IZAS); Roux et al. 2016: 350.

**Synonym**: *Lesticusdubius* Dubault, Lassalle & Roux, 2013: 209 (Type locality: Maoershan, Guangxi; Holotype in CDW). Roux et al. 2016: 346. **syn. n.**

**Chinese common name**: 壮劫步甲 (Zhuàng Jié Bù Jiă)

**Distribution.** Guangxi.

***Lesticustaiwanicus* Roux & Shi, 2011**

Roux and Shi 2011: 106 (Type locality: Fushan, Taiwan; Holotype in NMNS); Roux et al. 2016: 368.

**Chinese common name**: 台湾劫步甲 (Tái Wān Jié Bù Jiă)

**Distribution.** Taiwan.

***Lesticustristis* Roux & Shi, 2011**

Roux and Shi 2011: 108 (Type locality: Lancang, Yunnan; Holotype in IZAS); Roux et al. 2016: 354.

**Chinese common name**: 暗劫步甲 (Àn Jié Bù Jiă)

**Distribution.** Yunnan.

***Lesticusviolaceous* sp. n.**

**Chinese common name**: 紫光劫步甲 (Zĭ Guāng Jié Bù Jiă)

**Distribution.** Yunnan (Yingjiang).

***Lesticuswrasei* Dubault, Lassalle & Roux, 2013**

Dubault et al. 2013: 213 (Type locality: Tianbaoyan mt., Fujian; Holotype in CDW). Roux et al. 2016: 348

**Chinese common name**: 弗氏劫步甲 (Fú Shì Jié Bù Jiă)

**Distribution.** Fujian (Tianbaoyao).

***Lesticusxiaodongi* sp. n.**

**Chinese common name**: 晓东劫步甲 (Xiăo Dōng Jié Bù Jiă)

**Distribution.** Yunnan (Yingjiang).

## Supplementary Material

XML Treatment for Lesticus auripennis

XML Treatment for Lesticus bii

XML Treatment for Lesticus violaceous

XML Treatment for Lesticus xiaodongi

XML Treatment for Lesticus solidus
